# Myeloma Bone Disease: Update on Pathogenesis and Novel Treatment Strategies

**DOI:** 10.3390/pharmaceutics10040202

**Published:** 2018-10-24

**Authors:** Sonia Vallet, Julia-Marie Filzmoser, Martin Pecherstorfer, Klaus Podar

**Affiliations:** 1 Department of Internal Medicine, Karl Landsteiner University of Health Sciences, University Hospital, 3500 Krems an der Donau, Austria; martin.pecherstorfer@krems.lknoe.at (M.P.); klaus.podar@krems.lknoe.at (K.P.); 2 Karl Landsteiner University of Health Sciences, 3500 Krems an der Donau, Austria; 16KL100042@edu.kl.ac.at

**Keywords:** multiple myeloma, osteolytic bone disease, denosumab, bisphosphonates, Wnt inhibitors

## Abstract

Bone disease, including osteolytic lesions and/or osteoporosis, is a common feature of multiple myeloma (MM). The consequences of skeletal involvement are severe pain, spinal cord compressions, and bone fractures, which have a dramatic impact on patients’ quality of life and, ultimately, survival. During the past few years, several landmark studies significantly enhanced our insight into MM bone disease (MBD) by identifying molecular mechanisms leading to increased bone resorption due to osteoclast activation, and decreased bone formation by osteoblast inhibition. Bisphosphonates were the mainstay to prevent skeletal-related events in MM for almost two decades. Excitingly, the most recent approval of the receptor activator of NF-kappa B ligand (RANKL) inhibitor, denosumab, expanded treatment options for MBD, for patients with compromised renal function, in particular. In addition, several other bone-targeting agents, including bone anabolic drugs, are currently in preclinical and early clinical assessment. This review summarizes our up-to-date knowledge on the pathogenesis of MBD and discusses novel state-of-the-art treatment strategies that are likely to enter clinical practice in the near future.

## 1. Introduction

Multiple myeloma (MM) is the second most common hematological malignancy in adults with a median age of diagnosis of approximately 70 years. It accounts for 1% of all cancers, with a yearly mortality rate of 4.1/100,000 [1]. MM is characterized by bone marrow (BM) plasmacytosis and monoclonal protein in the blood and/or urine. Active myeloma is defined by the presence of one or more of the CRAB criteria—hypercalcemia (calcium >11 mg/dL), renal insufficiency (creatinine >2 mg/dL), anemia (hemoglobin <10 mg/dL), and bone lesions (≥1lesion on skeletal survey—determined using computed tomography (CT) or positron emission tomography (PET). In the absence of CRAB features, one or more of the following MM defining events is needed: plasma cell (PC) infiltration of ≥60% of the BM, free light chain ratio ≥100, and ≥1 magnetic resonance imaging (MRI) lesion ≥5 mm in size. These symptoms and signs are absent in premalignant stages of MM, including monoclonal gammopathy of undetermined significance (MGUS) and smouldering myeloma (SMM) [2].

Bone disease, comprising osteolytic lesions and/or diffuse osteopenia, is a common feature of MM. It occurs in up to 80% of patients and persists even in the absence of active disease, since complete repair of bone lytic lesions rarely occurs [3,4,5]. Indeed, MM is the malignancy with the highest percentage of metastatic bone disease. Myeloma bone disease (MBD) predominantly affects the axial skeleton (vertebrae (33%), ribs (15%), and sternum (13%)). MBD may lead to serious complications or skeletal-related events (SREs), defined as spinal cord compression, fractures, hypercalcemia, and the need for palliative surgery or radiation. As a result, patients´ quality of life is reduced due to severe pain, psychological distress, and loss of autonomy [6]. In addition, SREs are associated with a significant increase in mortality in MM patients [7,8].

MBD relies on an unbalanced bone remodeling, elicited by increased bone resorption mediated by osteoclasts (OC), and typically reduced bone formation due to the downregulation of the number of functional osteoblasts (OB) [9]. Functionally, MM cells interfere with physiologic bone remodeling by releasing OC-promoting cytokines such as receptor activator of NF-kappa B ligand (RANKL), interleukin (IL)-1, IL-6, chemokine C–C motif ligand 3 (CCL3), and CCL20. Moreover, MM cells are also responsible for the inhibition of osteogenesis, as they upregulate OB inhibitors including dickkopf-1 (DKK1) and sclerostin [10].

Imaging techniques to detect bone lesions significantly improved over the past few years. Compared to conventional skeletal survey (CSS), cross-sectional imaging methods, such as CT, MRI, and more recently fluoro-deoxyglucose (18 F-FDG) PET, allow for improved detection rate of bone lesions [11]. In a recent study, whole-body low-dose CT (WBLDCT) could detect lytic lesions in more than 25% of patients with negative CSS, leading to upstaging and changes in treatment plan [12]. In contrast to CT, MRI identifies bone marrow lesions without lytic reaction and effectively visualizes soft tissue masses extending from the bone. WBLDCT and MRI, therefore, replaced skeletal survey in the diagnosis and monitoring of MM and MBD in many institutions. Due to its ability to distinguish between metabolically active and inactive lesions, FDG PET is the preferred imaging modality to assess treatment response [13].

Therapeutic strategies, which target pathophysiologic interactions between MM cells, OCs, and OBs in the BM microenvironment, are crucial to delay the occurrence of SREs, to prevent bone lesions, and to attain tumor regression. Bisphosphonates (BP) and the recently approved RANKL inhibitor, denosumab, are bone-modifying agents (BMA) with anti-catabolic properties recommended for the treatment of MBD [14]. Other potential therapeutic targets include DKK1 and sclerostin antagonists [15]. In addition, classic anti-MM agents such as proteasome inhibitors (bortezomib and carfilzomib) and immunomodulatory drugs (IMiDs) have also an impact on lytic bone lesions by removing the driving force for MBD and by means of their anabolic and anti-catabolic properties, respectively.

Here, we summarize our current knowledge of MBD pathogenesis and its clinical management, with a special focus on the efficacy of BP and denosumab in preventing SREs and inhibiting MM proliferation, survival, and drug resistance. In addition, potential future therapeutic strategies for MBD are discussed.

## 2. Pathogenesis of Osteolytic Bone Disease in MM

### 2.1. Physiologic Bone Remodeling

The bone is a dynamic organ granting support and movement to the human body, and it is actively involved in hematopoiesis and endocrine functions. Bones are characterized by an elaborated network of marrow spaces and blood vessels within a matrix of hydroxyapatite and collagen, giving home to a heterogeneous cell population (bone cells, immune and endothelial cells, and mesenchymal and hematopoietic stem cells) [16]. The skeleton undergoes remodeling, a lifelong dynamic process of bone resorption and formation important to sustain the mechanical load, to preserve bone integrity and to maintain mineral homeostasis. Bone remodeling takes place in the basic multicellular unit (BMU), where OCs, OBs, and osteocytes work in a concerted and coordinated fashion. OCs and OBs are derived from different cellular lineages and possess opposite functions within the bone remodeling cascade [17].

OCs derive from the fusion of mononuclear cells of the monocyte–macrophage lineage into multinucleated active cells. They degrade the inorganic and organic bone matrix by binding tightly to the bone surface (sealing zone) and creating an acidic microenvironment rich in proteases (cathepsin and metalloproteinase) [18]. RANK, its ligand RANKL, and the decoy receptor osteoprotegerin (OPG) are considered key factors in regulating OC differentiation and activity [19].

OBs evolve from mesenchymal cells to osteocytes or bone-lining cells by going through specific differentiation steps modulated by time-dependent expression of transcription factors, such as Runt-related transcription factor 2 (RUNX2), Distal-Less Homeobox 5 (DLX5), and osterix, in a process called osteoblastogenesis [20]. Osteoblastogenesis depends on the balanced activity of agonists and antagonists of the Wnt signaling pathway, which regulates the expression of the transcription factor β-catenin. OBs secrete collagen and other extracellular structural as well as hormone proteins, such as osteopontin and osteocalcin, and they mineralize the bone matrix, thereby inducing bone formation [21]. They ultimately become bone-lining cells, inactive OBs laying on the bone surface; osteocytes, which are embedded in the mineralized matrix; or they undergo apoptosis. Osteocytes detect microcracks, mechanical strains, and changes in the hormonal milieu of the bone, and thus, trigger bone remodeling. Indeed, they play a key role in controlling the osteoclastic and osteoblastic activity, partly by secreting paracrine regulators of the remodeling process such as RANKL and the Wnt inhibitor sclerostin [22,23].

Changes in bone remodeling may be monitored by the detection of specific serum markers, including carboxy-terminal telopeptide of type-1 collagen (ICTP), β-crosslaps (CTX), and deoxypyridinoline (DPD), characteristic of bone degradation, as well as bone-specific alkaline phosphatase (BAP), osteocalcin, amino terminal pro-peptide of type I collagen (PINP), and carboxy terminal pro-peptide of type I collagen (PICP), characteristic of bone formation. Importantly, bone markers are also useful to monitor antiresorptive therapies and may help assessing fracture risk [24].

### 2.2. The Bone Niche in MM

Malignant plasma cells are home to the BM, whose cellular and extracellular microenvironments promote cell survival, tumor growth, and drug resistance, ultimately leading to MM progression and refractory disease. The cellular elements of the myeloma bone niche comprise bone marrow stromal cells (BMSCs), cells of the OB lineage, OCs, adipocytes, lymphocytes, and endothelial cells. Extracellular elements include the liquid milieu (growth factors, cytokines, and exosomes) and extracellular matrix (ECM) proteins such as osteopontin, collagen, and fibronectin.

Compared to normal cells, BMSCs of MM patients produce higher amounts of cytokines, such as IL-6, and induce T-lymphocyte dysfunction [25]. BMSCs modulate proliferation, migration, and drug resistance of MM cells also by releasing exosomes, small membranous vesicles that transfer oncogenic proteins, cytokines, messenger RNAs (mRNAs), and microRNAs to malignant plasma cells [26,27,28]. Integrin-mediated adhesion to BMSCs, as well as BMSC-secreted cytokines, stimulates tumor cell growth [29], at least, in part, via activation of the JunB transcription factor as recently shown [30]. Similarly, OCs provide proliferative and survival advantage to malignant plasma cells [31], in contrast to OBs that suppress tumor cell growth at least partly via the expression of decorin [32,33]. Interestingly, a recent study of the Croucher group suggests that OCs and OBs regulate cell dormancy and reactivation. Specifically, MM cells enter a quiescent non-mitotic state by interacting with bone-lining cells, whereas bone resorption induces MM cell proliferation [34].

Osteocytes and adipocytes play an emerging role as MM-promoting cells in the bone niche. Osteocytes induce MM cell growth via direct activation of the Notch signaling pathway as well as increase in Notch receptor expression [35]. Adipocytes, which are increased in the BM of MM patients, stimulate the proliferation and mediate the drug resistance of malignant plasma cells via secretion of adipokines, such as leptin and resistin. In addition, pre-adipocytes promote migration of MM cells [36,37,38,39].

In addition, immune cells, such as T lymphocytes, natural killer (NK) cells, and dendritic cells, populate the bone milieu. They are functionally defective in MM patients compared to healthy individuals and contribute to the immune escape of MM cells [40]. Moreover, the cluster of differentiation 4 (CD4)/CD8 T-cell ratio is decreased and immunosuppressive regulatory T cells (Tregs) are increased. Dysfunctional NK T-cells lack interferon-γ secretion [40,41]. Interestingly, recent studies demonstrated an upregulation of the pro-inflammatory T helper 17 (Th17) T-cell subset in MM patients, which contributes to the development of osteolytic lesions [42]. NK cell-mediated cytotoxicity is ineffective against MM cells, due to downregulation of natural cytotoxicity receptors (NCRs) and Natural Killer Group 2D (NKG2D) receptors expression, as well as upregulation of Programmed cell death protein (PD)-1surface levels on NK cells [43,44,45]. Finally, myeloid and plasmacytoid dendritic cells (DCs) are numerically decreased and functionally impaired in MM, thus further worsening T-cell dysfunction [46].

### 2.3. Myeloma-Associated Bone Disease

In cancer settings, an increase in OC activity disrupts the balance of the bone remodeling process leading to dramatic changes in the bone architecture and to the development of osteolytic lesions. In MM, in particular, effects of the upregulated osteoclastogenesis are amplified by the inhibition of OB activity and the increase in osteocyte apoptosis. In turn, bone cells regulate survival, proliferation, and drug resistance of malignant plasma cells, thus contributing to the vicious cycle of MBD. They also actively participate in engaging and disengaging tumor cells from dormancy and they may facilitate MM escape from the immune system [31,34,47].

Enhanced bone turnover is an early event in plasma cell disorders, also including premalignant diseases such as MGUS [48]. However, studies on bone biopsies from MGUS patients demonstrate regular OB activation for a balanced bone remodeling, whereas, in active MM, OC and OB activity is uncoupled with consequent disruption of the bone balance [49,50]. These pathogenetic events translate to changes in bone biomarkers, with an increase in parameters of bone resorption and suppression of bone formation markers detected in MM patients. By monitoring changes in the serum levels of markers of bone turnover, such as CTX and PINP, it is possible to detect disease progression and maybe select patients at risk of developing active MM [51,52].

**Figure 1:**
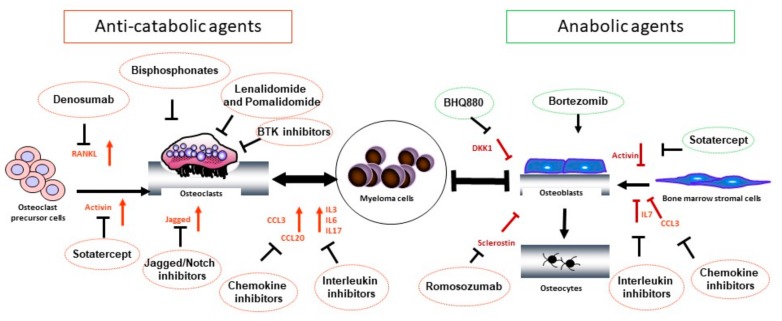
Schematic representations of the main signaling pathways involved in the pathogenesis of myeloma bone disease and their inhibitors. Malignant plasma cells modify their microenvironment by directly secreting and, indirectly, by stimulating the release of cytokines, which regulate osteoclastogenesis and osteoblastogenesis. Osteoclast-activating cytokines include RANKL, IL-3, IL-6, IL-17, CCL3, and CLL20. OB inhibition is mediated by MM-derived DKK1 and CCL3, as well as by BMSC-derived activin A and osteocyte-derived sclerostin. Importantly, MM cells also increase the RANKL/OPG ratio by stimulating osteocyte secretion of RANKL and inhibiting BMSC release of OPG. As a result of these complex interactions, the bone remodeling balance is disrupted and osteolytic lesions develop

Functionally, the increase in bone resorption in MM is due to an upregulation of signaling factors that promote OC differentiation and function, namely RANKL, chemokines (CCL3), and interleukins (IL-6). MM-derived exosomes, either from cell line or patient sera, also stimulate migration, survival, and differentiation of OC precursor cells [53]. In addition, tumor cells modify the surrounding microenvironment toward the inhibition of osteogenesis, by directly secreting Wnt antagonists such as DKK1 or by inducing the release of OB inhibitors from mesenchymal cells and osteocytes, such as sclerostin and activin. Indeed, recent studies suggest a key role for osteocytes in the development of MBD. The number of viable osteocytes is reduced in MM patients, especially in the presence of bone lesions due to increased apoptosis, and correlates with OC number [54]. Apoptotic osteocytes express high levels of RANKL and sclerostin, thus enhancing their ability to attract OC precursors and to inhibit OB differentiation (Figure 1) [35].

#### 2.3.1. Signaling Pathways Stimulating OC Activity in MM

##### RANK/RANKL Pathway

The RANK/RANKL pathway belongs to the most relevant physiologic and therapeutic signaling pathways for the regulation of bone resorption. RANK is a receptor of the tumor necrosis factor (TNF) superfamily, expressed on the surface of OC precursors. RANK ligand (RANKL) is the associated cytokine, secreted predominantly by osteocytes and, to a lesser extent, by BMSC and OBs [22]. By binding to RANK on immature OCs, RANKL induces their differentiation into mature cells [55]. OPG is a decoy receptor of the TNF superfamily, which also binds to RANKL. OPG is secreted by OBs and acts as a RANKL antagonist, thus inhibiting osteoclastogenesis.

In MBD, malignant plasma cells directly secrete RANKL and also stimulate its release via by T lymphocytes and osteocytes in the bone niche [56]. Specifically, MM cells induce osteocyte apoptosis via activation of Notch signaling, and apoptotic osteocytes express high levels of RANKL [35,54]. In addition, interactions of MM cells with the BM microenvironment lead to upregulation of different cytokines (IL-1 and TNF-α) and hormones (parathyroid hormone-related protein), which induce RANKL and decrease OPG expression, resulting in an increase of OC activity and bone destruction. Due to the critical role of OCs in MBD, suppression of OC maturation via RANKL blockade not only decreases bone resorption, but also inhibits tumor development in preclinical models of MM [57]. These data highlight the therapeutic relevance of targeting RANKL signaling in MM. 

##### Notch/Jagged Pathway

The canonical Notch pathway includes four receptors (Notch 1–4) and five ligands (Delta-like (DLL)1, 3–4, and Jagged 1–2). Following ligand binding, the intracellular domain of the receptor is released via protease (Tumor necrosis factor-alpha-converting enzyme (TACE/ADAM) and γ-secretase)-mediated cleavage and translocates to the nucleus to interact with several transcriptional factors [58,59]. Physiologic activation of the Notch/Jagged pathway regulates the survival of hematopoietic stem cells in the bone microenvironment partly via activation of the c-MYC transcription factor. Its aberrant activation contributes to the development of hematologic malignancies, and is critical for the progression and chemoresistance of MM [60]. Notch/Jagged signaling is also involved in osteolytic bone destruction via RANK/RANKL activation. By activating Notch on malignant plasma cells and OC precursor cells, BMSCs and MM cell-derived Jagged ligands stimulate RANKL expression in an autocrine and paracrine loop. In turn, RANK activation upregulates Notch 2 gene expression in OC precursors, thus potentiating RANKL signaling. Interestingly, Notch activation in osteocytes stimulates RANKL expression and increases the RANKL/OPG ratio. Due to its widespread expression in the MM microenvironment and its regulatory role on the RANK/RANKL pathway, Notch is a promising target in MM treatment [35,61].

##### Chemokines

Chemokines are small cytokines regulating cell migration during immune response and angiogenesis. In addition, they play an important role in the pathogenesis of MBD.

Chemokine (C–C motif) ligand 3 (CCL3), also known as macrophage inflammatory protein (MIP)1-α, is a pro-inflammatory cytokine secreted by MM cells and promotes autocrine migration and adhesion via binding to different receptors (such as chemokine C–C motif receptor 1 (CCR1) and CCR5). CCL3 is a strong osteoclastogenic factor, which promotes cell–cell fusion to form multinucleated OCs and stimulate RANKL expression by BMSCs [62,63,64]. Levels of CCL3 correlate with the extent of bone disease and bone resorption markers in MM [65].

CCL20 (MIP-3α) is a chemokine involved in the recruitment of T helper 17 (Th17) cells during inflammation and is also implicated in MM osteolytic disease. CCL20 is secreted by BMSCs, OBs, and OCs upon stimulation by MM cells, which also induce expression of its receptor CCR6 on OCs. Similar to CCL3, CCL20 induces osteoclastogenesis by increasing the number of precursor cells [66]. Levels of CCL20 correlate with the extent of MBD [66,67].

TNF-α is a proinflammatory cytokine involved in physiologic and pathologic processes, including rheumatoid arthritis and cancers. It promotes survival of MM cells by activating the nuclear factor kappa B (NF-κB) signaling pathway and by stimulating autocrine IL-6 production, and induces MM cell migration via upregulation of monocyte chemoattractant protein (MCP)-1 in tumor cells [68]. In addition, TNF-α acts synergistically with RANKL to stimulate osteoclastogenesis and inhibits OB differentiation by downregulating osterix transcription [69,70]. 

##### Interleukins

Interleukins are cytokines involved in immune regulation, inflammatory response, and hematopoiesis. In MM, several interleukins, such as IL-6, IL-3, IL-17, IL-1, and IL-11, regulate cell proliferation, survival, and drug resistance, and promote osteolytic lesions, thus underscoring the pathogenetic role of the deregulated immune system in MM.

IL-6 is secreted by BMSCs and OCs. In addition to representing a key survival factor for malignant plasma cells, it directly enhances OC differentiation and activation by binding to its receptor on OC precursor cells, and indirectly by upregulating osteopontin and vascular endothelial growth factor (VEGF) expression [71]. IL-3 derives from activated lymphocytes and stimulates CCL3 and RANKL-induced osteoclastogenesis and bone resorption. In addition, it induces activin A production, leading to increased osteoclastogenesis and decreased osteogenesis [72,73,74]. IL-17 expressed by T helper 17 lymphocytes has pro-osteoclastogenic properties. Of note, its levels correlate with the extent of bone disease in MM [75].

Similar effects on bone resorption and formation were described for IL-11 and IL-1β [76,77]. Interestingly, IL-11 expression in osteocytes of MM patients is higher than in healthy donors, correlating with the OC number [54].

#### 2.3.2. Signaling Pathways Suppressing OB Activity in MM

##### Canonical and Non-Canonical Wnt Pathways

The Wnt signaling pathway is important for several physiologic processes like embryogenesis, organ formation, bone remodeling, and insulin sensitivity. Canonical Wnt signaling is mediated by the transcription factor β-catenin. Briefly, in the absence of Wnt signaling, β-catenin is bound to a complex mediating its degradation by ubiquitination. However, by binding to the receptor Frizzled and co-receptor lipoprotein receptor-related protein (LRP)-5/6, Wnt activates Dishevelled (Dsh) which releases β-catenin from the destruction complex and allows it to translocate into the nucleus and initiate transcription [78,79,80]. 

Aberrant Wnt signaling was described in MM, where it is responsible for MM cell proliferation, migration, and adhesion-mediated drug resistance [81]. In addition, inhibitors of the canonical Wnt pathway such as sclerostin, DKK1, and secreted Frizzled-related protein (sFRP)2/3 are elevated in MM and inhibit bone formation by preventing β-catenin signaling [63,82,83]. 

Sclerostin is a glycoprotein produced by osteocytes in response to mechanical strain of the body. The lower the mechanical strain is, the higher the sclerostin secretion becomes. Sclerostin binds to LRP4, which acts as a chaperone presenting sclerostin to the Wnt co-receptors LRP5/6, thus facilitating inhibition of Wnt/βcatenin signaling [84,85]. Ultimately, sclerostin impairs OB differentiation and bone mineralization; it also induces apoptosis of mature OBs by caspase activation, and stimulates osteoclastogenesis by increasing the RANKL/OPG ratio [23,86]. MM cells induce osteocyte apoptosis, which correlates with increased expression of RANKL and sclerostin [35]. Indeed, increased levels of sclerostin are demonstrated in MM patients with evidence of bone fractures at diagnosis [83,87].

DKK1 is another antagonist of the Wnt signaling pathway secreted by MM cells [88]. By binding to LRP6, it inhibits osteoblastogenesis and new bone formation. DKK1 is also responsible for enhanced sclerostin secretion in the bone microenvironment, since sclerostin is released by immature OBs in the presence of MM-derived DKK1 [83]. Moreover, DKK1 increases the RANKL/OPG ratio, which stimulates osteoclastogenesis. Interestingly, inhibition of Wnt signaling increases the secretion of IL-6 that stimulates proliferation of MM cells, and thus, enhances DKK1 release in a vicious cycle [89]. High DKK1 gene expression levels correlate with MBD and may predict for SRE development in MM patients undergoing bisphosphonate treatment [82,90].

MM cells also inhibit osteogenic differentiation of BMSCs by interfering with the non-canonical Wnt5a signaling pathway. Specifically, they reduce the expression of the co-receptor Ror2 in preOBs, thus preventing their differentiation in mature cells [91].

##### Activin A

Activin A is a member of the transforming growth factor beta (TGF-β) family of proteins produced by many cell types throughout development. It signals through SMAD2/3 proteins to regulate a variety of functions, including cell proliferation, differentiation, apoptosis, wound healing, and metabolism.

In MM patients, elevated serum levels of activin A correlate with lytic bone lesions and advanced disease stage. Indeed, activin A inhibits OB differentiation and promotes osteoclastogenesis in MM. Malignant plasma cells upregulate activin A secretion by BMSCs. It exerts its inhibitory effects on OBs by downregulating the transcription factor DLX5 in precursor cells of the OB-lineage [92]. In addition, activin A promotes OC differentiation via non-canonical signaling, namely activation of the NF-κB pathway in a RANKL-independent fashion [93].

##### Chemokines and Interleukins

As soluble inhibitors of osteogenesis in MM, IL-7 and CCL3 contribute to the development of MBD. MM-derived IL-7 is responsible for the inhibition of OB formation via RUNX2 downregulation [94]. CCL3, in addition to its osteoclastogenic activity, represses OB function via osterix downregulation. An early increase in CCL3 levels was detected in animal models of MM, and they correlated with reduced mineralization and bone formation at early time points, despite normal OB counts [95,96]. 

## 3. Treatment of Osteolytic Bone Disease in MM

Treatment of MBD relies on the use of BMAs, which alleviate the complications of skeletal lesions and reduce the occurrence of SREs. Bisphosphonates represented, until recently, the standard of care for MBD. Importantly, in January 2018, the anti-RANKL antibody, denosumab, was approved in MM for the same indications as BPs. Both agents inhibit bone resorption by targeting OCs and differ mainly in terms of their impact on renal function [97]. Based on the recent progress of our understanding of the MBD pathogenesis, several new agents with a broad range of mechanisms of actions are under development. Importantly, classic anti-MM agents, proteasome inhibitors, and IMiDs also show effects on the bone, and combination strategies of MM- and bone-targeting compounds represent a promising treatment approach in active MM (Figure 1) [10,15,98].

### 3.1. Bisphosphonates

As derivates of pyrophosphates, BPs tightly bind to hydroxyapatite crystals in the bone, where they are absorbed by OCs. They exert their anti-catabolic effect by inhibiting OC formation and differentiation, as well as by inducing OC apoptosis. BPs not retained in the skeleton are rapidly cleared from circulation by renal excretion. Importantly, they also display anti-tumor and immunomodulatory activity [99]. Their chemical structure correlates with their strength: nitrogen-containing BP, like pamidronate (PAM) or zoledronic acid (ZOL), are 100 to 10,000 times more potent compared to non-nitrogen containing BP (such as clodronate) [100].

The most widely used BPs in MM are PAM and ZOL, both approved as monthly intravenous administration for patients with active MM, either with lytic lesions or with osteoporosis in the absence of osteolysis. PAM and ZOL show comparable effects in reducing the incidence of SREs, and they are more effective than oral agents such as clodronate (CLO) for SRE prevention. Importantly, based on the results of the Medical Research Council (MRC) Myeloma IX trial, ZOL not only significantly prevents SREs in MM patients with and without lytic bone disease, but also improves progression-free, as well as overall, survival compared to CLO [101]. BPs are also recommended for pain control resulting from osteolysis or in fracture management as adjunctive treatment to radiotherapy, surgery, and analgesics. Additional indications for BPs include the presence of osteoporosis in low- and intermediate-risk asymptomatic MM or in MGUS.

The main side effects of BPs are renal toxicity and osteonecrosis of the jaw (ONJ); therefore, patients undergoing long-term treatment should be closely monitored. PAM induces glomerular lesions, while ZOL induces acute tubular necrosis, both associated with acute renal failure [102]. Regular surveillance of serum creatinine and urinary proteins is, thus, important during treatment [103]. ONJ is a potentially serious condition affecting 1–10% of the patients in long-term treatment, with higher incidence with ZOL than PAM (10% versus 4%). ONJ in patients receiving BPs is defined by the presence of exposed bone in the jaw lasting for more than eight weeks, despite proper dental management. Severity and symptoms are variable; some patients experience pain and fistulae, whereas a few may even remain asymptomatic. Risk factors for ONJ are tooth extraction or other invasive dental surgeries, long duration of BP therapy, poor oral hygiene, and older age. To prevent ONJ all patients should receive dental review and appropriate treatments before starting BP therapy [104]. Rare complications associated with long-term BP use are atypical fractures of the femoral shaft, which occur with minimal trauma and predominantly affect young patients [105,106]. They may result from BP-mediated suppression of bone remodeling, leading to accumulation of microdamages and impaired damage repair [107,108].

Due to long-term toxicity, BP treatment should be limited to two years for MM patients in remission. In addition, a three-monthly schedule should be considered for patients with stable disease or on maintenance therapy. Indeed, the bone-marker-directed ZOL administration evaluated in the recent Zoledronic Acid - Bone MARKer-Directed Dosing (Z-MARK) study shows that less frequent ZOL dosing (every 12 weeks over two years) is associated with two-year ONJ incidence of 3.3%, while maintaining a low SRE rate (Table 1) [109].

### 3.2. Denosumab

RANKL is a key player in the pathogenesis of MBD, and its inhibition via the monoclonal anti-RANKL antibody, denosumab, is an effective treatment strategy in MM. Denosumab binds to RANKL with high affinity and specificity; it inhibits its interaction with RANK and results in supressed bone resorption [110]. Phase 1 clinical trials showed that single and multiple applications of denosumab in healthy postmenopausal women led to sustained suppression of OC-mediated bone resorption [111]. In phase 2 studies in solid cancers and advanced MM, denosumab resulted in a significant inhibition of SREs [112,113]. Denosumab was approved for the treatment of osteoporosis as twice yearly subcutaneous applications, and for bone metastases in solid cancers as monthly injections in 2010.

The efficacy of denosumab compared to ZOL in MM patients was evaluated in two large randomized phase 3 clinical trials. The “244” study enrolled more than 1700 patients with solid cancers (except breast and prostate cancers) and MM. It demonstrated the superiority of denosumab compared to ZOL in terms of time to first on-study SRE (20.6 versus 16.3 months), with a comparable toxicity profile. Interestingly, the overall survival data in the MM subgroup (300 patients) were inferior in the denosumab arm compared to the ZOL arm [114]. The unbalance in baseline variables (lower Eastern Cooperative Oncology Group (ECOG) performance status and International Staging System (ISS) stage in the ZOL group) and treatment strategies (less transplantation in the denosumab arm) may at least, in part, explain the observed difference in survival [115]. A follow-up study enrolling only MM patients stratified for therapy and ISS stage was, therefore, launched. The “482” study included 1718 patients, 1:1 randomized to denosumab or ZOL. The primary endpoint of non-inferiority to ZOL for time to first SRE was met. Grade 3 adverse events and ONJ incidence were infrequent and similar between treatment arms. Renal toxicity was reported in 10% of patients in the denosumab group versus 17% in the ZOL group; hypocalcemia was more frequent in the denosumab arm (17% versus 12%) [14]. Intriguingly, preliminary data suggest a progression-free survival advantage for denosumab compared to ZOL; further studies to confirm these results are ongoing (Table 1) [116].

Based on the results of this study denosumab was approved in January 2018 for the treatment of MM patients with active disease and as additional pain control management in the case of lytic lesions or fractures. Treatment recommendations to prevent ONJ do not differ from BPs. Due to the favorable renal toxicity profile, denosumab is recommended in patients with compromised renal function. As for treatment duration of denosumab therapy, there are no specific recommendations; caution is advised in the case of abrupt treatment interruptions, since denosumab has a reversible mechanism of action.

### 3.3. Anti-Tumor Therapies with Bone-Modifying Effects

#### 3.3.1. Proteasome Inhibitors

Proteasome inhibition emerged as a backbone treatment strategy in MM, exerting its anti-tumor activity directly by promoting cell apoptosis and indirectly by modifying the bone microenvironment [117]. Of note, the proteasome pathway also plays an important role in OB differentiation. Its inhibition by bortezomib stimulates OB activity and number, partly by upregulating the RUNX2 transcription factor, with a consequent increase in bone formation [118]. In addition, bortezomib inhibits osteoclastogenesis and OC bone resorption activity [119]. In MM patients, bortezomib-based regimens increase the number of RUNX2-positive OBs and lead to a significant rise in osteoblastic markers, specifically BAP and osteocalcin [120,121]. Importantly, an increase in bone formation markers is only observed in responding patients and is transient. After reaching a peak in the 6th week of treatment, BAP levels slightly decline [120,122]. Moreover, bortezomib increases the number of viable osteocytes, partly by inhibiting MM-induced autophagy and apoptosis of osteocytes [123].

#### 3.3.2. Immunomodulatory Agents

IMiDs, such as lenalidomide and pomalidomide, represent other therapeutic backbones for MM treatment. IMiDs induce MM cell apoptosis, increase anti-MM T- and NK-cell immunity, and inhibit angiogenesis. In vitro studies on pomalidomide and lenalidomide suggest inhibition of osteoclastogenesis via downregulation of PU.1, a key factor for OC differentiation [124,125]. In addition, treatment with IMiDs normalizes the RANKL/OPG ratio in MM [126]. Patients responding to lenalidomide and dexamethasone regimens have a decline in bone resorption markers, but no change in markers of bone formation. On the contrary, adding bortezomib to the combination leads to a decrease in bone resorption and an increase in bone formation markers, independently of treatment response. These effects are at least, in part, due to the normalization of the RANKL/OPG ratio and a reduction in DKK1 levels, respectively [127].

### 3.4. Wnt Pathway Modulators

Despite the key role of OB suppression in MBD, currently approved treatment strategies have only anti-catabolic properties. The clinically most advanced anabolic strategies involve inhibitors of the Wnt pathway, namely DKK1 and sclerostin antagonists. 

Specifically, neutralizing antibodies, such as BHQ880 and MabB3, and DNA-based vaccines were developed to target DKK1 [128,129,130]. BHQ880 is a fully human immunoglobulin G1 (IgG1) anti-DKK1 antibody, which stimulates OB differentiation and inhibits myeloma cell growth via alteration of the BM microenvironment [128,131]. A phase 2 clinical trial with high-risk SMM patients demonstrated bone anabolic effects of single agent BHQ880, without significant anti-tumor activity [132]. BHQ880 in combination with ZOL and anti-myeloma treatments was well tolerated and increased bone mineral density in relapsed or refractory MM patients [133].

The main source of DKK1 in MM is malignant plasma cells, and, since not all patients express DKK1, there may be differences in response to anti-DKK1 therapies. In contrast, sclerostin is expressed by osteocytes and its inhibition may be a more effective therapeutic strategy. Treatment with romosozumab, a humanized monoclonal antibody against sclerostin, enhanced bone mineral density in osteoporotic patients, leading to its approval for the treatment of osteoporosis [134]. Preclinical studies in MM showed that genetic inhibition of sclerostin prevents MBD development in early MM models, and anti-sclerostin antibodies reduce osteolysis and increase bone mass in advanced MM models, without having an impact on tumor burden [83,87,135]. Since sclerostin inhibition has no significant anti-tumor activity, combination strategies may be critical to achieve anti-MM and bone-protecting effects. Indeed, the combination of anti-sclerostin with the new proteasome inhibitor, carfilzomib, results in tumor burden reduction [83,136].

### 3.5. Promising Agents in Preclinical and Early Clinical Development

Based on pathogenetic studies several new agents are under preclinical and clinical evaluation, among them, inhibitors of activin A, the Notch pathway, chemokines, and interleukins, as well as epigenetic therapies (Table 2).

Inhibition of activin A by a decoy receptor effectively reduced skeletal lesions and decreased tumor burden in animal models of MBD by reversing OB inhibition [92,137]. In a phase 2 clinical trial, treatment of newly diagnosed or relapsed/refractory MM patients with the activin A fusion receptor, sotatercept, in combination with standard chemotherapy (melphalan, dexamethasone, and thalidomide), improved bone mineral density and bone formation compared to placebo. In addition, a positive effect on anemia was observed [138]. Interestingly, based on in vitro studies, sotatercept may be effectively combined with lenalidomide, which stimulates activin A secretion via activation of the JNK pathway. The combination demonstrates anabolic and anti-tumor activity in preclinical models of MBD [139].

Anabolic effects were also observed using epigenetic therapies. MM cells induce permanent repressive epigenetic changes at the *Runx2* promoter of mesenchymal cells, thus suppressing OB differentiation. Inhibition of Histone deacetylase (HDAC)1 activity in OB precursor cells reverses this effect and rescues osteoblastogenesis [140]. Similarly, the HDAC inhibitor, vorinostat, promotes OB differentiation by upregulating the transcription factor RUNX2. In a murine model of MM, treatment with vorinostat and quisinostat prevents bone loss and development of osteolytic lesions [141,142]. Combination strategies with HDAC inhibitors are currently being evaluated in clinical trials. 

Considering the wide range of functions of the Notch signaling pathway in the pathogenesis of MM, its inhibition is considered a promising therapeutic strategy. In addition to reducing MM cell migration and growth, inhibition of Notch via γ-secretase inhibitor (GSI) XII impairs OC differentiation and demonstrates in vivo anti-MM and anti-catabolic effects [143,144]. Despite the encouraging preclinical data with GSI inhibitors, severe gastrointestinal toxicity caused by simultaneous inhibition of Notch 1 and 2 receptors may preclude their further clinical development [145]. Strategies to mitigate these side effects are based on intermittent dosing schedules and use of glucocorticoids [146]. In addition, antibody-based targeting of Notch receptors or ligands represents a valid alternative to pan-Notch inhibitors, due to their promising anti-tumor activity and better tolerability [147,148].

The promiscuity in ligand–receptor interactions of chemokines is a challenge for their clinical development, since each receptor may have a distinct role in MM pathogenesis. However, preclinical data indicate that CCR1 may be a promising target for MBD [149]. CCR1 inhibition via a small molecule exerts a strong anti-catabolic effect by inhibiting OC formation and function, thus reducing bone osteolytic lesions in animal models [62,150]. In addition, it overcomes CCL3-induced OB inhibition. Animal models further confirmed this dual effect of CCR1 antagonists by demonstrating upregulation of osteocalcin expression along with OC downregulation [95].

Similar inhibitory effects on OCs were shown with an anti-human IL-17A antibody, which additionally impairs MM cell survival [151]. IL-6 is another interesting target. A fully humanized monoclonal antibody against IL-6 (1339) demonstrated anti-tumor activity, as well as inhibition of bone resorption, in mouse models of MBD as a single agent and synergistic effects with conventional anti-MM agents [152]. Finally, promising agents with anti-MM and bone modifying effects are the inhibitors of the Bruton’s tyrosine kinase (BTK). BTK belongs to the B-cell antigen receptor signaling pathway, regulates B-cell development, and participates in the progression of B-cell malignancies. Indeed, BTK inhibitors are approved treatment strategies in lymphoma. Interestingly, the BTK pathway is also activated by RANKL signaling in OCs, and its inhibition by ibrutinib leads to a decrease in OC number and bone resorption activity in vitro and in animal models of MBD [153].

## 4. Concluding Remarks

Despite therapeutic improvements, more than 40% of MM patients suffer from SREs, and new treatment strategies are, therefore, needed. Skeletal disease and related complications are associated with significant morbidity and mortality rates in MM. In addition to bisphosphonates, which represented the standard of care for MBD during the last two decades, the RANKL inhibitor, denosumab, was approved in January 2018 for patients with active MM, providing a safe alternative to bisphosphonates in case of compromised renal function. Importantly, results of the MRC IX and “468” trials indicate that treatment with BMAs provides a survival advantage for patients with active MM [101,116]. The pathogenesis of bone disease in MM depends on OC activation, as well as on the inhibition of OBs and osteocytes. As a result, the balance of bone remodeling is irreversibly disrupted leading to defective bone repair. A major challenge in the treatment of MBD is to revert bone damage. Despite disease remission, conventional MM chemotherapies (i.e., melphalan and doxorubicin) are unable to completely heal lytic bone lesions [3]. However, recent studies suggest that proteasome inhibitors, in particular bortezomib, may promote bone repair via their anti-tumor and anabolic activities [4,5]. Bortezomib-induced bone sclerosis occurs in 20% to 72% of patients with osteolysis, depending on the line of treatment. Bone repair is independent from anti-MM response level and is heterogeneous, since only a small fraction of patients show signs of sclerosis in all lytic lesions. Based on these data, ongoing research revolves predominantly around agents which stimulate new bone formation, such as Pim2 kinase inhibitors which are currently being investigated in relapsed/refractory MM patients (NCT01456689) [154]. In addition, ongoing studies are evaluating the effect on bone turnover of novel anti-MM agents, including the proteasome inhibitor, ixazomib (NCT02499081), and the anti-CD38 antibody, daratumumab (NCT03475628). The ultimate goal is to restore a balanced bone remodeling, thereby not only improving MBD, but also reducing tumor burden, slowing down disease progression, and reverting bone damage.

## Figures and Tables

**Table 1:** Zoledronate and denosumab in myeloma bone disease.

	Zoledronate	Denosumab
Agent	Nitrogen-containing bisphosphonate	Fully human anti-RANKL IgG2 monoclonal antibody
Indications [97]	Patients treated for active myeloma with or without lytic lesions.	Myeloma patients with evidence of lytic bone lesions
Dosing schedule [97,109]	Renal-adapted, iv administration every 3–4 weeks or every 12 weeks	sc administration, every 4 weeks
Suggested duration of treatment [97]	Up to 2 years	No recommendations available
Median time to first SRE [14]	24 months	22.8 months
Median PFS [116]	35.4 months	46.1 months
Renal toxicity [14]	17%	10%
ONJ [14]	3%	4%
Hypocalcemia [14]	12%	17%
Monitoring	- Serum creatinine (before each administration) - Albuminuria (every 3–6 months) - Serum calcium, vitamin D, phosphate, and magnesium (on a regular basis) - Dental examination (before first administration and on a regular basis)	- Serum calcium, vitamin D, phosphate, and magnesium (on a regular basis) - Dental examination (before first administration and on a regular basis)

Abbreviations: RANKL, receptor activator of NF-kappa B ligand; IgG2, immunoglobulin G2; iv, intravenous; sc, subcutaneous; SRE, skeletal-related event; PFS, progression-free survival; ONJ, osteonecrosis of the jaw.

**Table 2:** Agents in early clinical and preclinical development.

Molecular Target	Function	Therapeutic Relevance
Jagged/Notch pathway [35,61]	Jagged derives from MM cells and BMSC Notch activation in tumor cells, OC precursors and osteocytes stimulates RANKL secretion Notch activation in osteocytes leads to cell apoptosis	Notch inhibition via γ-secretase inhibitor (GSI) XII has anti-MM effects and inhibits OC differentiation, thus improving bone architecture in animals models of MM [143,144]
CCL3 (MIP-1α) [62,63,64]	CCL3 is secreted by MM cells It attracts OC precursors inducing cell multinucleation It stimulates RANKL expression by BMSCs CCL3 inhibits OB maturation	Inhibition of the CCL3 receptor CCR1 has anti-catabolic effects and stimulates OB activity in MBD models [62,95]
CCL20 (MIP-3α) [66]	CCL20 derives from BMSC, OB, and OC in response to MM cells CCL20 stimulates osteoclastogenesis	
IL-3 [72,73,74]	IL-3 derives from activated lymphocytes It amplifies the osteoclastogenic effect of CCL3 and RANKL It induces activin A production	
IL-17 [75]	IL-17 is expressed by Th17 cells It stimulates osteoclastogenesis	Anti-IL17A antibody inhibits OC differentiation, and decreases tumor growth and bone lesions in animal models of MM [151]
IL-7 [94]	IL-7 downregulates RUNX2, thus inhibiting OB differentiation	
IL-6, IL-1β, IL-11 [71,76,77]	IL-6, IL-1β, and IL-11 stimulate OC differentiation IL-6 upregulates osteopontin and VEGF expression, which induce OC activity	IL-6 mAb (1339) shows anti-tumor activity and inhibits bone resorption in animal models of MBD [152]
DKK1 [83,88]	DKK1 is secreted by MM cells It inhibits OB differentiation It stimulates secretion of sclerostin and IL-6 It increases RANKL secretion	DKK1 inhibition stimulates OB differentiation and reduces IL-6 levels in vitro Anti-DKK1 mAb restore bone formation and inhibit tumor growth in preclinical models [128,131] BHQ880 has bone anabolic effect alone in smouldering MM patients [132] BHQ880 in combination with ZOL and anti-MM therapy increases bone mineral density in MM patients [133]
Sclerostin [23,86]	Sclerostin derives from osteocytes It suppresses osteoblastogenesis and mineralization, and induces apoptosis of mature OBs It increases RANKL/OPG ratio	Sclerostin inhibition prevents MBD and reduces osteolysis in preclinical models MM Sclerostin inhibition in combination with carfilzomib reduces tumor burden and inhibits bone disease in animal models of MM [83]
Activin A [92,93]	Activin A is released by BMSCs under MM cell influence It downregulates DLX5 expression in OB precursor, thus preventing cell differentiation It promotes OC differentiation via non-canonical NF-κB pathway activation in precursor cells	Activin A inhibition via a decoy receptor reverses bone lesions and decreases tumor burden in MBD models [92,137] Activin A inhibition together with lenalidomide has a strong anti-tumor and anabolic activity in animal models [139] Sotatercept (ACE-011) in combination with anti-MM therapy has bone anabolic effect [138]

Abbreviations: CCL, chemokine C–C motif ligand; MIP, macrophage inflammatory protein; DKK1, dickkopf-1; MM, multiple myeloma; OC, osteoclast; BMSCs, bone marrow stromal cells; OB, osteoblast; IL, interleukin; Th, T-helper lymphocytes; mAb, monoclonal antibody; NF-κB, nuclear factor kappa B; VEGF, vascular endothelial growth factor; ZOL, zoledronate; MBD, myeloma bone disease.
